# Lymphoma patients treated with anti-CD20 and chemotherapy display disconnected T and B cell responses to COVID-19 vaccine

**DOI:** 10.3389/fimmu.2024.1524813

**Published:** 2025-01-20

**Authors:** Sylvain Lamure, Houria Hendel Chavez, Marie-Ghislaine de Goër de Herve, Luc-Matthieu Fornecker, Bernard Drenou, Caroline Jacquet, Fatiha Merabet, Milena Kohn, Florence Quélin, Angela Jackson, Sylvain Choquet, Rémy Duléry, Yassine Taoufik, Caroline Besson

**Affiliations:** ^1^ Centre de Recherche en Cancérologie de Lyon, Centre Léon Bérard, Lyon, France; ^2^ Service d’onco-hématologie, Hôpitaux Nord-Ouest, Villefranche-Sur-Saône, France; ^3^ Laboratoire Institut national de la santé et de la recherche médicale (INSERM) U1186, Institut Gustave Roussy, Villejuif, France; ^4^ Service d’Hématologie, Institut de Cancérologie Strasbourg Europe, Strasbourg, France; ^5^ Service d’Hématologie, Groupe Hospitalier de Mulhouse Sud Alsace, Mulhouse, France; ^6^ Service d’Hématologie, Centre Hospitalier Régional Universitaire (CHRU) de Nancy, Nancy, France; ^7^ Service d’Hématologie Oncologie, Centre Hospitalier de Versailles, Le Chesnay, France; ^8^ Maison de la Recherche Clinique, Centre Hospitalier de Versailles, Le Chesnay, France; ^9^ Université Paris-Saclay, Université de Versailles – Saint-Quentin-en-Yvelines (UVSQ), Inserm, Équipe “Exposome et Hérédité”, Centre de Recherche en Epidémiologie et Santé des Populations (CESP), Villejuif, France; ^10^ Pitie-Salpetriere Hospital, Assistance Publique - Hôpitaux de Paris (APHP)- Sorbonne University, Paris, France; ^11^ Service d’Hématologie Clinique et de Thérapie Cellulaire, Hôpital Saint Antoine, Assistance Publique - Hôpitaux de Paris, Sorbonne Université, Inserm UMRs 938, Paris, France

**Keywords:** COVID-19 vaccine, lymphoma, immune response, anti-CD20, chemotherapy

## Abstract

Due to immunosuppressive treatment, COVID-19 vaccination is challenging in patients with B-cell lymphoma. We prospectively evaluated CD4, CD8 T-cell and serological responses to the COVID-19 mRNA vaccine in a cohort of patients treated for a B-cell lymphoma with anti-CD20 therapy. During lymphoma treatment, CD4, CD8, and CD19 cell dropped. While functional-specific CD4 and CD8 T-cell responses to SARS-CoV-2 were unaffected, vaccination in patients on treatment induced low specific antibody titers, contrasting with a preserved serological response when vaccination was completed before treatment initiation. Those findings reinforce a vaccinal strategy based on completion before lymphoma treatment, with a booster administered afterward.

## Introduction

B-cell lymphoma patients were initially identified at high risk for death or a severe course of COVID-19, especially for those treated with B-cell-depleting immunotherapies (1). The incidence of death due to COVID-19 decreased from 30% to 6% early in 2022 (2) with the spreading of the omicron strain and the use of antiviral drug combinations such as nirmatrelvir/ritonavir, convalescent plasma, and the generalization of vaccination (3). Vaccination is challenging among patients treated for a B-cell malignancy: administering anti-CD20 monoclonal antibodies alone or combined with T cell-depleting agents like cyclophosphamide and bendamustine is immunosuppressive. In addition, T-cell exhaustion is a common feature in the context of cancer. Different studies showed that repeated vaccine injections improved the seroconversion rate and antibody titer (4) and that the CD4 T cell response is not impaired by the malignancy and its treatments (5–8). Regarding CD8 cytotoxic T-cell (CTL), few studies have been conducted on this population, but one reported a preserved CTL response (9).

We designed the Lymphocovac study to address the impact of B-cell lymphoma treatment on the functional cellular and humoral immune response to the COVID-19 mRNA vaccine and determine the optimal vaccination time.

## Methods

We ran a prospective multicentric study (NCT 05050461) between November 2021 and July 2022, including adult patients with B-cell malignancy either during lymphoma therapy or after treatment completion, vaccinated against SARS-COV2 with at least two shots of a commercial mRNA vaccine. Patients treated with CAR T-cells and/or allogeneic transplantation, as well as those with a short life expectancy (i.e., six months or less), were excluded. We also recruited a cohort of healthy individuals who received at least two shots of the same vaccine among patients’ spouses.

We collected data regarding vaccination (number of injections, dates), clinically and biologically documented episodes of COVID-19 and their severity, monoclonal antibody prophylaxis, lymphoma characteristics and treatments (subtype of lymphoma, anti-tumoral treatment combination, date of treatment initiation, date of treatment completion and treatment response).

A 45 mL peripheral blood sampling was performed 3 to 9 months after the last vaccine injection. Flow cytometry was used to determine the percentages and absolute counts of CD4 T cells, CD8 T cells, CD19+ B-cells, and CD16+ CD56+ natural killer (NK) cells counts (BD Multitest), and the expression of the exhaustion markers CTLA4, PD1, TIM3, LAG3, and TIGIT on CD4 and CD8 T cells (BD LSR Fortessa). Euroimmun ELISA Quantivac test was used to determine SARS-CoV-2 antibody titer; the positivity threshold was defined for titers higher than 11 relative units (RU/mL). To determine CD4 and CD8 T-cell responses, Interferon-gamma (IFN-g) and Tumor Necrosis Factor-alpha (TNF-a) secretion by CD4 T cells and Perforin and Granzyme B secretion by CD8 T cells were assessed by intracellular flow cytometry after overnight incubation of cells with overlapping peptide pools covering SARS-CoV-2 immunodominant sequences (SARS-CoV-2 Select Peptivator, Miltenyi Biotec). The background observed in non-activated cells was deducted to evaluate the specific anti-SARS-CoV-2 response. Results were indicated as IFN-g and/or TNF-expressing cells per mm3 for CD4 T cells and perforin and/or granzyme B-expressing cells per mm3. Flow cytometry data were analyzed with FlowJo Software.

Quantitative variables are presented with their median. We categorized immunological and clinical data according to the time interval since their last treatment [cut-off at 12 months between the categories "treatement" and "post-treatment" as anti-CD20 induced immunodepression lasts up to 12 months (10)]. Comparisons were made with the Mann–Whitney test or Fisher’s exact test. Analysis and figures were made with GraphPad software version 9.

## Results

We included 49 individuals, 42 B-cell lymphoma patients, and 7 healthy spouses. Patients characteristics are presented in **Table 1**: 60% were male, and the median age was 66 (IQR 38-77). All patients received at least 2 injections of mRNA vaccine, and 64% had no history of clinically or biologically documented COVID-19. 48% are suffering from DLBCL, 31% from follicular lymphoma, and 60% of them received a single line of treatment. All of them received an anti-CD20 therapeutic monoclonal antibody; the last treatment was a combination with chemotherapy for 74% of patients. 21% of them had prior bendamustine exposure.

**Table 1:** Patient’s characteristics.

Patients Characteristics	N = 42
Male N, (%)	25 (60)
Age (median, IQR)	66 (38-77)
Vaccination	
2 shots, n (%)	8 (19)
3 shots, n (%)	32 (76)
4 shots, n (%)	2 (5)
COVID Infections	
0, n (%)	27 (64)
1, n (%)	15 (36)
Post Vaccination COVID infection, n (%)	4 (10)
Lymphoma histology	
DLBCL, n (%)	20 (48)
FL, n (%)	13 (31)
MZL/LPL, n (%)	8 (19)
MCL, n (%)	1 (2)
Lymphoma treatment (ttt)	
1 line, n (%)	25 (60)
2 lines, n (%)	8 (19)
3 lines, n (%)	9 (21)
Bendamustine based regimen, n (%)	9 (21)
Anti CD20 + chemotherapy (last ttt), n (%)	31 (74)
Anti CD20 monotherapy (last ttt), n (%)	11 (26)

IRQ, interquartile range; DLBCL, diffuse large B cell lymphoma; FL, follicular lymphoma; MZL, marginal zone lymphoma; LPL, lymphocplasmacytic lymphoma; MCL, mantle cell lymphoma.

**Figure 1:**
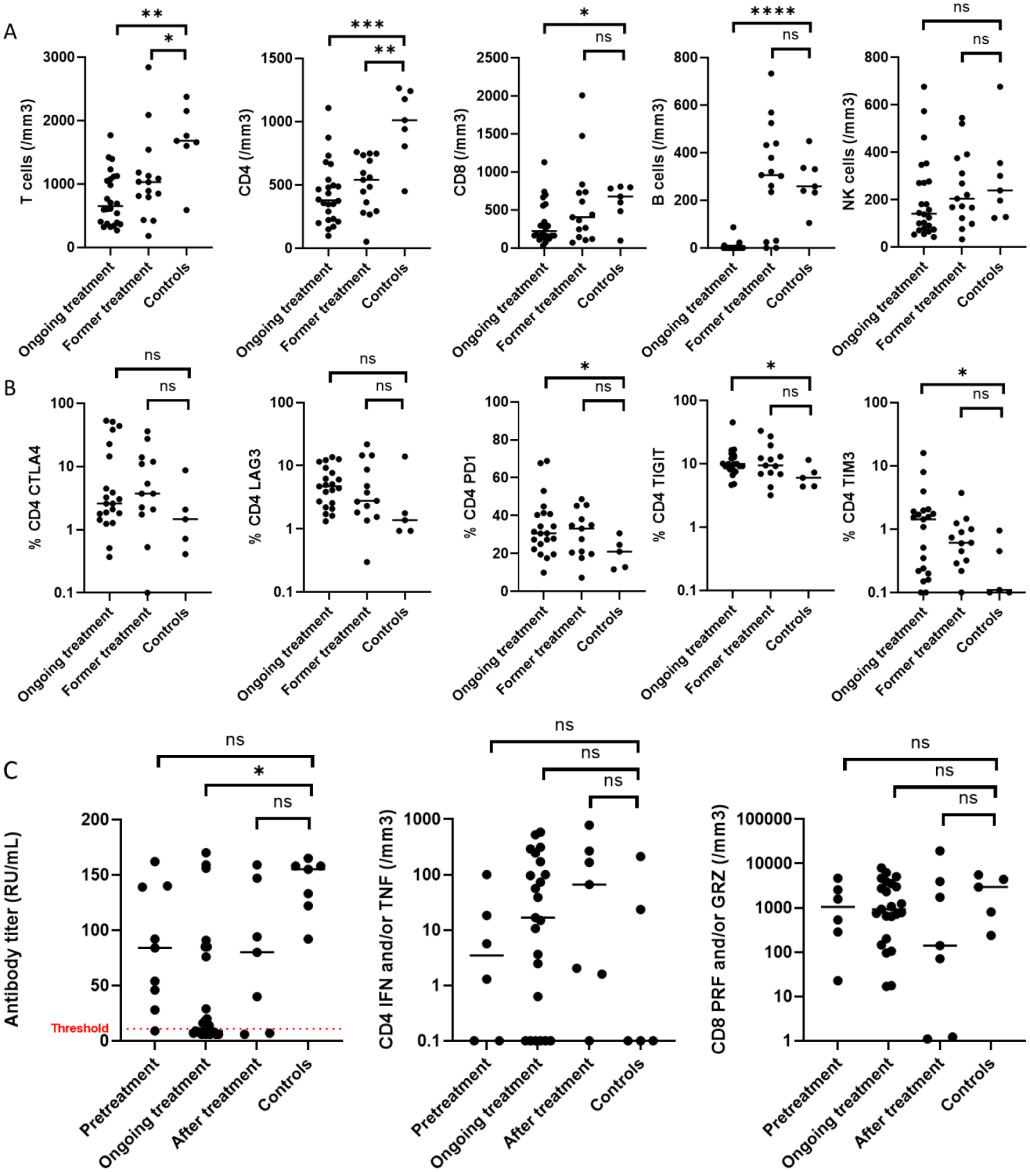
Immune deficiency and vaccinal response in B-cell lymphoma. 7 healthy controls were compared to 42 patients according to their treatment status: ongoing treatment when sampling was performed during treatment (between initiation and up to 1 year after its completion), or former when treatment was completed more than a year before the sampling, **(A)** for their T, CD4 and CD8, B and NK cell count, **(B)** for the exhaustion’s markers of CD4 T cells. **(C)** compares the vaccinal response: antibody titer, with a positivity threshold at 11 RU/mL, CD4 expressing interferon (IFN) and/or tumor necrosis factor (TNF) and CD8 expressing perforin (PRF) and/or granzyme B (GRZ) of patients that were fully vaccinated before starting their treatment, during treatment or within 1 year after its completion, or more than 1 year after treatment completion, to healthy individuals. Mann-Whitney test was performed using Prism Graph Pad, p-value summary: *0.01-0.05, **0.001-0.01, ***0.001-0.0001, ****<0.0001: ns, not significant.

We compared lymphocyte counts of patients with healthy individuals (**Figure 1A**). CD4 T cell counts were higher in healthy individuals (median count 1.01 G/L) than among patients: a recent treatment had a stronger impact (median count 0.38 G/L, p<0.01) than an older treatment (0.54 G/L, p <0.01). Similarly, CD8 counts (median 0.22 G/L) of recently treated patients were lower than healthy individuals (0.68 G/L, p=0.03). As expected, B cells were undetectable in patients recently treated (0.26 G/L in healthy donors, p< 0.01). B cell reconstitution occurred after 12 months after treatment completion. Treatment did not impact NK cell count.

Then, we measured the expression of exhaustion markers. CD4 T cells of recently treated patients had more exhaustion features than those treated longer ago, with a higher expression of PD1 (31% vs. 21%, p=0.04), TIM3 (1.1% vs. 0.1%, p=0.04) and TIGIT (9.9% vs. 6%, p=0.03), there was a trend for LAG3 (4.7% vs. 1.4%, p=0.08), but similar expression of CTLA4 (**Figure 1B**). Furthermore, CD8 expression of TIM3 that was higher for patients undergoing treatment (p=0.05) and other exhaustion markers on CD8 was similar in patients and healthy controls (**Supplementary Figure S1**).

Blood was sampled between 10 and 426 days after the last injection. Time from last vaccination did not correlate with humoral or cellular response; except in the subgroup of controls where median antibody titer decreased over time (r -0.8, p=0.03), and CD4 response tended to decrease (p=0.05), contrary to the CD8 response. We analyzed vaccinal response according to the timing of the last vaccine shot: before the start of lymphoma treatment, during treatment, until 12 months after treatment completion, or more than 12 months after treatment completion, and compared to healthy individuals (**Figure 1C**). Patients who received 2 shots had similar serological and cellular responses to those receiving 3.

Patients serological response was lower than healthy controls (all seropositive) when vaccination was performed during treatment (13/25 seronegative, p=0.02), and similar to those of controls when vaccination was completed either before its initiation (1/9 seronegative), or more than a year after completion (2/7 seronegative). Considering only seropositive individuals, median antibody titer of healthy individuals was 155 RU/mL (IQR 128-158), compared to 84 RU/mL for patients vaccinated before treatment start (IQR 46-139, p=0.07), 20 RU/mL for patients vaccinated during treatment (IQR 8-76, p=0.03) and 80 RU/mL for patients vaccinated after treatment (IQR 24-121, p=0.25).

In contrast, we found no difference between groups in CD4 production of interferon and/or tumor necrosis factor and in CD8 production of perforin and/or granzyme B. Of note, a high expression of TIM3 on CD4 cells correlated with a lower antibody titer (Spearman r score -0.37, p=0.02), as well as a high TIGIT expression on CD4 correlated with a lower number of CD8 perforin and/or granzyme B positive (Spearman r score -0.35, p=0.04) (**Supplementary Figure S2**). We observed no difference in vaccine response according to bendamustine use or anti-CD20 monotherapy or combination therapy (**Supplementary Figure S3**).

We identified 19 individuals with a documented infection: 15 cases of SARS-Cov-2 infection occurring before vaccination, and 4 cases of SARS-Cov-2 infection occurring post-vaccination. The 15 infections occurred before vaccination in 4 healthy controls and in 11 patients, of which 2 had a severe COVID-19 requiring hospitalization. Antibody titers, CD4 and CD8 T cell responses of the individuals infected before their vaccination were similar to those with no prior documented infection. Among the 4 patients that were infected post-vaccination, none of them required hospitalization or oxygen supply. The median time between the last injection and infection was 126 days (min 25, max 220); 3 were suffering from DLBCL, one from follicular lymphoma, 2 had no detectable neutralizing antibodies, the others had 92 and 170 RU/mL (median 50 RU/mL) at the time of the study, 3 of them had a functional CD4 response. All of them had a functional CD8 response. The 4 patients were vaccinated between the start of the treatment and a year after completion.

## Discussion

In this prospective case-control study, we measured the impact of B-cell lymphoma treatments on the functional immune response to SARS-CoV-2 mRNA vaccination. B cell depletion and lower CD4 and CD8 counts were found in patients under treatment or recently treated for lymphoma. We also observed exhaustion features on CD4 T cells. Patients vaccinated between lymphoma treatment initiation and one year after its completion showed a poor antibody response but preserved CD4 and CD8 T cell responses, contrasting with a preserved B cell vaccinal response when vaccination was completed before the treatment initiation.

Indeed, long lived vaccinal virus-specific memory B cells as well as long lived plasma cells can resist to rituximab treatment (11), preserving vaccinal protection acquired before anti-CD20 initiation. To our knowledge, this study is the first to report a preserved memory response to mRNA SARS-CoV-2 vaccines for some patients treated with anti-CD20.

Previous works reported an impaired humoral response in patients with B-cell lymphoma that could be counteracted with a booster dose (4, 5, 12). Different studies showed a preserved CD4 cellular immune response in patients treated with chemotherapy (7, 8, 13) or CD19-CAR T-cells (14). Regarding CTL response, a study (9) reported comparable amounts of CD8 that recognize HLA class 1 combined with SARS-CoV-2 peptides in patients recently treated for lymphoma. Here, we used a more functional approach measuring the perforin and granzyme response to Spike Protein. CD8 and B cell responses require functional CD4 T-cells (15), and we detected a high frequency of exhaustion markers expressed on CD4 T cells during antitumor treatment, suggesting a dysfunctional state that may affect the humoral response after vaccination in some cases.

In this cohort, we did not observe any specific impact of bendamustine or the use of anti-CD20 alone versus in combination with chemotherapy agents, which may be due to a small sampling. Despite a heterogeneity in the timing of vaccinal response evaluation, we found no significant effect of time since last vaccination on response in patients. Similarly, the occurrence of a COVID-19 infection had no impact on serological and cellular response in this cohort.

Taken together, those results confirm that the cellular helper and cytotoxic response is preserved but that the serological response is poor when the vaccination program is performed during or just after antitumor treatment. They argue for a vaccination program completed before its initiation and a booster injection after its completion.

## Data Availability

The raw data supporting the conclusions of this article will be made available by the authors, without undue reservation.
