# Supplementary material for: Lymphoma patients treated with anti-CD20 and chemotherapy display disconnected T and B cell responses to COVID-19 vaccine
Source: Front Immunol. 2025 Jan 20;15:1524813. doi: 10.3389/fimmu.2024.1524813 (PMC11788169; doi:10.3389/fimmu.2024.1524813)
Supplement: Supplementary file 1 [file SupplementaryFile1.pdf]

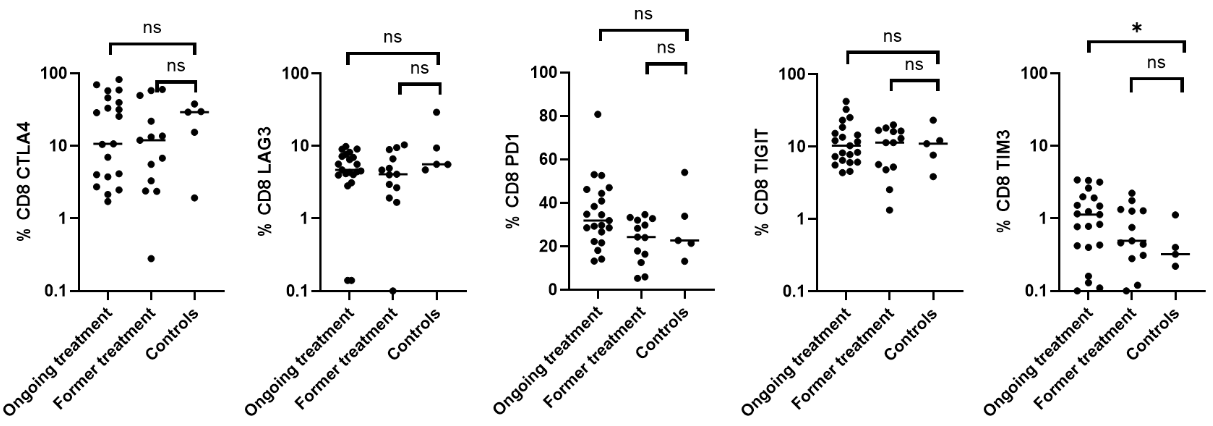

<u>Supplementary Figure 1. Exhaustion markers expressed on CD8 T cells.</u> 7 healthy controls were compared to 42 patients according to their treatment status: ongoing treatment when sampling was performed during treatment (between initiation and up to 1 year after its completion), or former when treatment was completed more than a year before the sampling, for the exhaustion's markers of CD8 T cells. Mann-Whitney test was performed using Prism Graph Pad, p-value summary: *0.01-0.05.

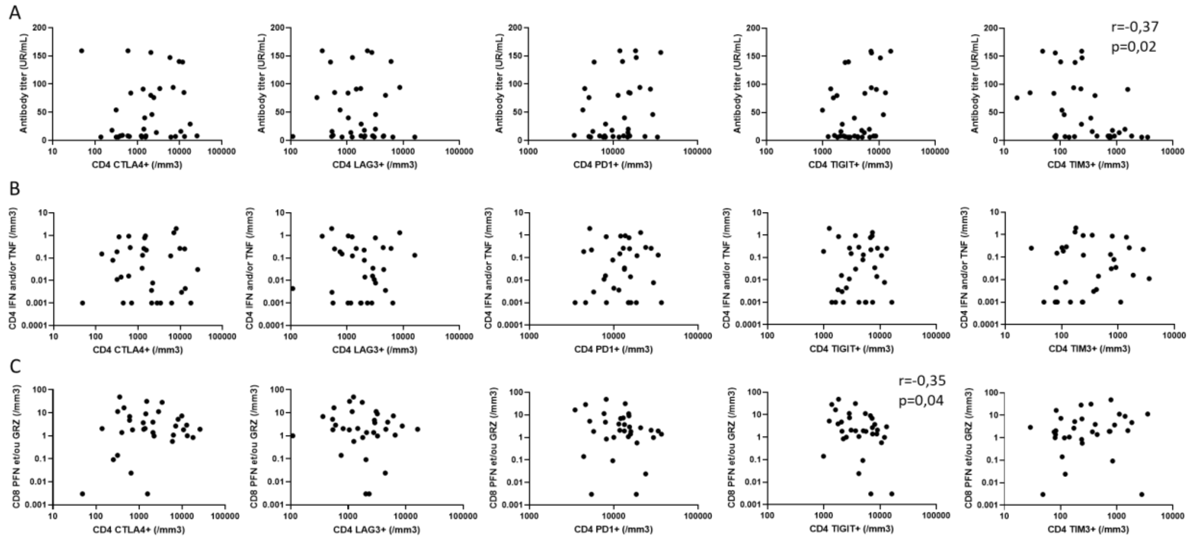

<u>Supplementary Figure 2. Immune response as a function of CD4 T cell exhaustion.</u> The level of expression of the different co-inhibitory receptors CTLA4, LAG3, PD1, TIGIT and TIM3 was correlated with (A) the antibody titer, (B) the count of CD4 expressing interferon (IFN) and/or tumor necrosis factor (TNF) and (C) the count of CD8 expressing perforin (PRF) and/or granzyme B (GRZ). Spearman 's correlation was performed using Prism Graph Pad.

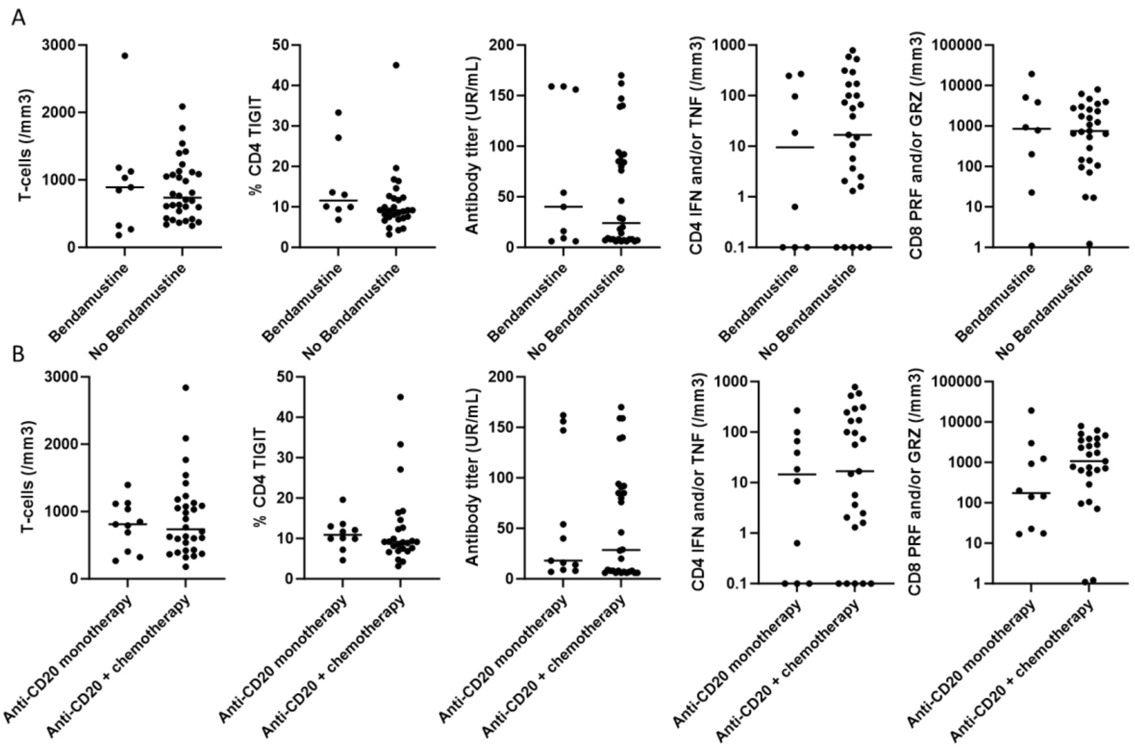

<u>Supplementary Figure 3. Immune response according to treatment modality.</u> T cell count, CD4 TIGIT expression, antibody titer, with a positivity threshold at 11 UR/mL, CD4 expressing interferon (IFN) and/or tumor necrosis factor (TNF) and CD8 expressing perforin (PRF) and/or granzyme B (GRZ) response to COVID-19 vaccine are compared according to treatment modality (A) previous use of bendamustine (B) last anti-CD20 monoclonal antibody administered alone or in combination with any chemotherapy. Mann-Whitney test was performed using Prism Graph Pad.
